# Intravital imaging of the murine subventricular zone with three photon microscopy

**DOI:** 10.1093/cercor/bhab400

**Published:** 2022-01-14

**Authors:** Bin Sun, Mengran Wang, Anna Hoerder-Suabedissen, Chris Xu, Adam M Packer, Francis G Szele

**Affiliations:** Department of Physiology, Anatomy and Genetics, University of Oxford, Oxford OX1 3PT, UK; School of Applied and Engineering Physics, Cornell University, Ithaca, NY 14853, USA; Department of Physiology, Anatomy and Genetics, University of Oxford, Oxford OX1 3PT, UK; School of Applied and Engineering Physics, Cornell University, Ithaca, NY 14853, USA; Department of Physiology, Anatomy and Genetics, University of Oxford, Oxford OX1 3PT, UK; Department of Physiology, Anatomy and Genetics, University of Oxford, Oxford OX1 3PT, UK

**Keywords:** 3-photon, microscopy, mouse, neurogenesis, subventricular zone

## Abstract

The mouse subventricular zone (SVZ) produces neurons throughout life. It is useful for mechanism discovery and is relevant for regeneration. However, the SVZ is deep, significantly restricting live imaging since current methods do not extend beyond a few hundred microns. We developed and adapted three-photon microscopy (3PM) for non-invasive deep brain imaging in live mice, but its utility in imaging the SVZ niche was unknown. Here, with fluorescent dyes and genetic labeling, we show successful 3PM imaging in the whole SVZ, extending to a maximum depth of 1.5 mm ventral to the dura mater. 3PM imaging distinguished multiple SVZ cell types in postnatal and juvenile mice. We also detected fine processes on neural stem cells interacting with the vasculature. Previous live imaging removed overlying cortical tissue or lowered lenses into the brain, which could cause inflammation and alter neurogenesis. We found that neither astrocytes nor microglia become activated in the SVZ, suggesting 3PM does not induce major damage in the niche. Thus, we show for the first time 3PM imaging of the SVZ in live mice. This strategy could be useful for intravital visualization of cell dynamics, molecular, and pathological perturbation and regenerative events.

## Introduction

The subventricular zone (SVZ) is a specialized neural stem cell (NSC) niche lining the lateral ventricles. It has been extensively characterized at the cellular level and is a robust system for neurodevelopmental mechanism discovery (Ihrie and Alvarez-Buylla 2011; Llorens-Bobadilla et al. 2015; Sun et al. 2018). SVZ neurogenesis consists of sequential cell functions, including NSC activation, transit amplifying progenitor (TAP) cell proliferation, neuroblast migration, and differentiation. The NSCs exhibit apical basal polarity with an apical primary cilium contacting the CSF and a basal process contacting blood vessels. The SVZ is also important for perinatal brain development, as one of the main sources of forebrain glia (Levison and Goldman 1993) and olfactory bulb (OB) neurons (Altman 1969). This stem cell niche can be stimulated to increase neurogenesis, which limits neuronal injury and neurodegeneration (Young et al. 2011). In contrast to this beneficial role, SVZ stem cells can be tumorigenic; for example, transformation by the IDH1^R132H^ mutation in the SVZ leads to gliomagenesis (Bardella et al. 2016).

The SVZ has not been visualized in intact live animals, with the greatest hurdle being its deep location. Brain tissue is highly heterogeneous and causes unacceptable scattering of short two-photon wavelength photons. Nevertheless, two-photon microscopy (2PM) imaging of ex vivo slice cultures has been useful in analyzing neuroblast migration in the SVZ and rostral migratory stream (RMS) (Nam et al. 2007; James et al. 2011; Martinez-Molina et al. 2011). We previously showed that rostral neuroblast migration was interspersed with local exploratory movements, periods of immobility, dorsoventral, and caudal migration. However, differences in the culture media and in vivo extracellular fluid can affect NSC proliferation and fate choice and it is unclear how accurately slice culture reflects other cell dynamics in vivo (Silva-Vargas et al. 2016).

Intravital two-photon microscopy (2PM) is typically restricted to a depth of approximately 700 microns of tissue, but the SVZ and subgranular zone (SGZ) are more than twice as deep. Therefore, others have used cortical windows and 2PM in the brains of live mice to image the SGZ stem cell niche (Pilz et al. 2016, 2018). One study generated excellent data over two months and followed individual clones of cells as they expanded and differentiated (Pilz et al*.* 2018). Whereas powerful insights into neurogenic niche biology can be gathered with this approach, it can be argued that it is in fact a lesion model, a type of traumatic brain injury. We and others have shown that cortical lesions ranging from thin needle stabs to wide aspiration lesions activate neurogenic niches and parenchymal astrocytes (Szele and Chesselet 1996; Buffo et al. 2008; Chang et al. 2016).

Another approach is coupling of 2PM with gradient index (GRIN) lenses, lowered into the live brain (Murray and Levene 2012). GRIN lenses have recently been used to rapidly image behaviorally relevant changes in SGZ neural activity were used to show that increasing neurogenesis inhibits activation of neurons by social stress (Anacker et al. 2018). GRIN lens approaches caused moderate inflammation and the development of a thin gliotic scar around the lens, which is not surprising given that the lenses were three hundred and fifty microns in diameter (Lee et al. 2016) and up to one thousand microns deep (Barretto et al. 2011). Potential imaging- or GRIN lens-induced inflammation in or around neurogenic niches must be approached cautiously as inflammation can be rapid and long lasting and is well known to alter neurogenesis (Ekdahl et al. 2003; Monje et al. 2003; James et al. 2016; Bardella et al. 2018).

To overcome these limitations, we developed the use of three-photon microscopy (3PM) to perform non-invasive imaging using lasers that pump photons at long wavelengths of 1700 nm, which significantly reduces the photon scattering problem. 3PM achieves unprecedented penetration depth in imaging biological tissues by combining improved nonlinear confinement and reduced tissue scattering at long wavelengths (Horton et al. 2013; Ouzounov et al. 2017; Wang, Wu, et al. 2018b). In this method, a fluorophore is excited by the simultaneous absorption of three photons of infra-red wavelength. As the wavelength is three times longer than that needed for conventional (single photon) fluorescence excitation, it can lie in the range where scattering is much lower. 3PM also exhibits high resolution, improves signal to background ratio, has sufficient frame rate for rapid biological events, and is compatible with a wide range of fluorophores. However, it was unclear if 3PM could be used to study the deep subcortical SVZ with adequate resolution to explore cell diversity during lineage progression. Here, we show that with 3PM the cellular morphologies of different cell types were successfully identified in the SVZ, to a maximum depth of ~1.5 mm.

## Materials and Methods

### Fluorescent Dye Preparation

CellTracker Orange (C34551), CellTracker Red (C34552), CellTracker Green (C7025) and CellTracker CM-DiI (C7000) were purchased from Life Technologies. CellTracker Orange, Red and Green were dissolved in 100% DMSO (Sigma) to a final concentration of 10 mM. CellTracker CM-DiI was dissolved in DMSO to the final concentration of 2 mM. 0.1% Fast Green was added to dyes for tracing ventricular injection when necessary.

### Mice and Brain Injection

Wild-type (CD1) mice were used for dye injections into postnatal mice (3 to 8-day-old) and wild-type (C57BL/6J) mice were used for dye injections into juvenile mice (5 to 8-week-old). Nestin-CreERT2 (Lagace et al. 2007) and Ai9 (007909) lines were purchased from the Jackson Laboratory. In Nestin-CreERT2^+/−^;Ai9^+/−^ mice, tamoxifen (Sigma, 150 mg/kg) was injected intraperitoneally once daily for five consecutive days. The postnatal lateral ventricle injection was performed as previously published (Sun et al*.* 2018). In brief, the pups were anesthetized by hypothermia. 0.5–1 μL fluorescent dye was injected and the pups recovered in a 37 °C warming box before return to the dam. For juvenile mouse lateral ventricle injections, the mice were first anesthetized with isoflurane and 2 μL fluorescent dye was injected. The stereotactic coordinates from Bregma were as follows: −0.75 mm (AP), 1.2 mm (ML), and −1.9 mm (DV) (Beckervordersandforth et al. 2010). A craniotomy window was performed for imaging and a glass window affixed. For SVZ imaging, the window encompassed the area of the skull surrounded by the sagittal, coronal, and lambdoid sutures. For OB imaging, the nasal bones were removed to create the windows across the midline. Animal procedures were reviewed and approved by the Cornell Institutional Animal Care and Use Committee. All animal procedures were carried out with Oxford University Research Ethics Committee approval in accordance with the Animals (Scientific Procedures) Act of 1986 (UK). Excluding surgical complications, imaging was successful in 8/8 pups and due to variations in the craniotomy in 5/10 juvenile mice at Cornell University. 3-photon imaging was successful in 25/25 postnatal pups at the University of Oxford.

For our Oxford experiments, the scalp was removed bilaterally from the midline to the temporalis muscles, and a metal headplate with a 5 mm circular imaging well was fixed to the skull overlying the SVZ with dental cement (Super-Bond C&B, Sun-Medical). A 4–5 mm circular craniotomy was performed during which any bleeding was washed away with sterile external solution or staunched with Sugi-sponges (Sugi, Kettenbach). Cranial windows composed of 4 or 5 mm circular glass coverslips were press-fit into the craniotomy, sealed to the skull by a thin layer of cyanoacrylate (VetBond) and fixed in place by dental cement.

### 3PM Imaging

Most of the experiments in this paper were performed in Cornell, except for those described in Figure 5, which were performed in Oxford. The Cornell setup consisted of a wavelength-tuneable optical parametric amplifier (OPA, Opera-F, Coherent) excitation source pumped by a femtosecond laser with a master oscillator power amplifier (MOPA) architecture (Monaco, Coherent) operating at 1650 nm. A silicon wafer is used to compensate for the dispersion of the optics of the light source and the microscope including the objective (Horton and Xu 2015). The laser repetition rate is maintained at 333 kHz with a maximum power of 55 mW under the objective lens. We used a 60–70 fs pulse width. The maximum average power used for the deepest imaging was below 55 mW with 1700 nm excitation, which is within the range of permissible average power considering the imaging depth and FOV. The images were acquired with a custom-built multiphoton microscope with a high-numerical aperture objective (Olympus XLPLN25XWMP2, 25X, NA 1.05). Two detection channels were used to collect the fluorescence signal and the third harmonic generation (THG) signal by photomultiplier tubes (PMTs) with GaAsP photocathodes (H7422-40, Hamamatsu). For RFP imaging at 1650 nm, we used a 593 nm long-pass filter (FF01-593/LP-25, Semrock) for fluorescence and a band-pass 562/40 nm filter (FF01-562/40-25, Semrock) for THG collection. For signal sampling, the PMT current is converted to voltage and low-pass filtered (20 kHz) by a transimpedance amplifier (C7319, Hamamatsu). Analog-to-digital conversion is performed by a data acquisition card (NI PCI-6110, National Instruments). The signal acquisition system displayed shot-noise limited performance, and light shielding was carefully done to achieve dark counts of 20–40 photons per second under actual imaging conditions without laser scanning. ScanImage 3.8 (Vidrio Technologies) running on MATLAB (MathWorks) was used to acquire images and control a 3D translation stage to move the sample (MP-285, Sutter Instrument Company). All imaging depths and thickness are reported in raw axial movement of the motorized stage. High-resolution structural images were typically taken with 512 × 512 pixels/frame, 0.24 Hz frame rate, and multiple frame averages at each depth.

The Oxford setup consisted of a wavelength-tunable optical parametric amplifier (OPA, Mango SP, Amplitude) pumped by a femtosecond laser (1030 nm, Satsuma HP2, Amplitude) operating at 1300 nm. A double-pass dual prism compressor is used to compensate for the dispersion of the optics of the light source and the microscope including the objective, achieving a pulse width of 47 fs under the objective. The laser repetition rate is maintained at 1 MHz with a maximum power of 100 mW under the objective lens. The images were acquired with a galvo scanning multiphoton microscope (VivoScope, Scientifica UK) and a high-numerical aperture, long working distance objective (Olympus XLPLN25XSVMP2, 25X, 1.0 NA, 4 mm working distance). One PMT (H10770PA-40, Hamamatsu) detection channel was used to collect green fluorescence using a dichroic (T565LPXR, Chroma) and bandpass filter (ET525/50m-2p, Chroma). The signal was then converted to voltage by a transimpedance amplifier (150 kOhm, 1 MHz; XPG-ADC-PREAMP) and digitized (NI-5734, National Instruments). ScanImage 2021 (Vidrio Technologies) running on MATLAB (MathWorks) was used to acquire images. A motorized stage (Scientifica) was used to control the position of the sample and a focusing motor (Scientifica) was used to control the position of the objective and PMT detection apparatus relative to the sample. All imaging depths are reported in raw axial movement of the motorized stage. High-resolution structural images were typically taken with 1024 × 1024 pixels per frame at 3.2 μs dwell time (0.27 Hz) and an average of eight frames at each depth.

### Immunohistochemistry and Confocal Imaging

Immunohistochemistry was performed as previously published (Sun et al*.* 2018). In brief, 3PM imaged mice were perfused and brains were fixed with 4% PFA. Brains were sectioned coronally at 30 μm with a microtome and immunohistochemistry carried out on free-floating sections. Sections were blocked with PBS+ (10% donkey serum, 0.1% Triton-X in PBS) and incubated with primary antibodies overnight at 4 °C, followed with Alexa fluor-conjugated secondary antibodies (Invitrogen). The following primary antibodies were used: rat anti-GFAP (1:500, Invitrogen 130300), goat anti-Dcx (1:100, Santa Cruz sc-8066), goat anti-Iba1 (1:500, Abcam ab5076), rabbit anti-S100ß (Dako Z031129-2), mouse anti-Mash1 (1:100, BD Biosciences 556604). DAPI (Sigma) was used for counterstaining cell nuclei blue before mounting. Zeiss confocal scanning microscopes (Model 710 or 800) were used for image acquisition. About, 1 μm intervals were used for Z-stack scanning.

### Image Analysis

All images were processed with ImageJ and Imaris (Bitplane). For cell type identification based on morphology, images were filtered with ImageJ 3D suite (Median).

## Results

**Figure 1:**
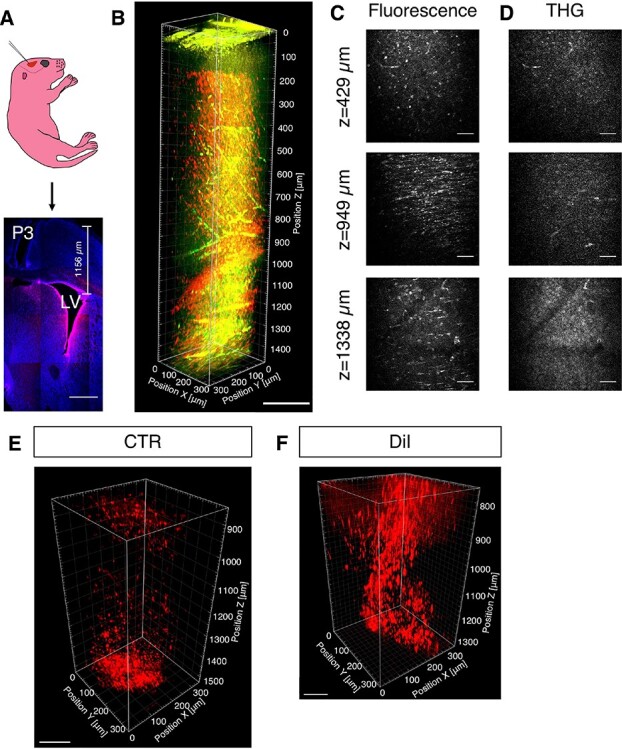
Validation of 3PM in postnatal SVZ imaging. (*A*) A schematic of LV injection in postnatal day 1 (P1) CD1 pup and tiled confocal imaging of a representative brain section labeled with CTO (red) and DAPI (blue), 2 days post injection (P3). (*B*) 3D reconstruction from 0 to 1472 μm below the pial surface of pup with CTO (3 dpi; red, CTO fluorescence; green, THG). (*C*, *D*) Selected XY frames at different depths in (*B*) (see Supplementary Movie 1). (*E*) 3D reconstruction of white matter and SVZ of pups injected with CTR (3 dpi). (*F*) 3D reconstruction of white matter and SVZ of pups injected with DiI (5 dpi). Scale bars represent 500 μm in *A*, 200 μm in *B*, 50 μm in *C* and *D*, 100 μm in *E*; 80 μm in *F*.

We used a custom-built 3PM (Supplementary Fig. 1*A*). The excitation source was a wavelength-tuneable optical parametric amplifier (OPA, Opera-F, Coherent) operating at 1650 nm to match the excitation wavelength of the fluorescent dyes used in this study. The spectra of the excitation source were measured by an Optical Spectrum Analyzer (OSA205C, Thorlabs), shown in Supplementary Figure 1*B*. Bandpass filters were added in the detection paths to separate third-harmonic generation (THG) signal from fluorescence signal (Fig. 1*D*). THG is generated at the interface of materials with different third-order susceptibility (Barad et al. 1997). In this study, we used THG to locate corpus callosum (CC) white matter depth as the SVZ lies immediately below the CC. CellTracker Orange (CTO) was injected into the lateral ventricle (LV) of P0-P1 pups under anesthesia and imaging was performed 3 days post injection (dpi) (Fig. 1*A*). We acquired *Z*-stacks approximately 1500 μm deep (Fig. 1*B*–*D* and Supplementary Movie 1). CTO fluorescence signals were found throughout the stack, but predominantly below 1000 μm, which was also confirmed with post-imaging histology (Fig. 1*A*). Other fluorescent dyes tested included CellTracker Red (CTR) (Fig. 1*E*) and DiI (Fig. 1*F*), and similar results were obtained.

Another capability of 3PM is imaging through un-thinned, intact mouse skull (Wang, Ouzounov, et al. 2018a). Our previous experience showed that the skull of 8–14 weeks old mice significantly attenuates the excitation beam. Although high resolution, high-contrast 3-photon imaging is possible through the intact skull, the penetration depth is less than 50% of that with cranial windows, which makes imaging of the juvenile or adult SVZ impossible. On the other hand, the skull of postnatal pups is thinner and likely to be more transparent than that of older mice, and eliminating the cranial window will open the new possibility of following the development of pups over extended periods of time. In our limited trials (*N* = 8 total, Cornell and Oxford), unfortunately, we were unable to detect convincing SVZ signals in pups with either through-skin or through-skull imaging (data not shown). Further investigation is needed for through-skull imaging of postnatal pups to ascertain such possibilities.

**Figure 2:**
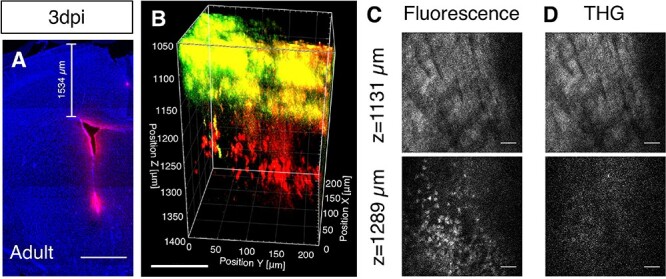
Validation of 3PM in juvenile SVZ imaging. (*A*) Confocal imaging of a representative juvenile brain section labeled with CTO (red) and DAPI (blue) at 3 dpi. (*B*) 3D reconstruction of white matter (THG) and CTO-labeled SVZ from the depth of 1050–1400 μm in a juvenile mouse (3 dpi; red, CTO fluorescence; green, THG) (see Supplementary Movie 2). (*C*, *D*) Selected XY frames at different depths in (*B*). Scale bars represent 1000 μm in *A*, 100 μm in *B*, 30 μm in *C* and *D*.

Compared to postnatal brains, juvenile brains contain mature myelinating oligodendrocytes in the corpus callosum and are therefore more light scattering. To confirm SVZ visualization, we injected CTO into the LV of 5- to 8-week-old mice. CTO fluorescence was detected around the LV as well as in the corpus callosum (CC), resulting from the injection and diffusion of the dye into adjacent regions (Fig. 2*A*, and Supplementary Movie 2). The THG signal in the CC was more distinguishable in these juvenile mice than in postnatal pups and extended from ~1000 to ~1153 μm in depth (Fig. 2*D*). The CC was characterized by longitudinal arrays corresponding to axon bundles. Below the CC, we found clearly labeled CTO+ cells (Fig. 2*B*–*D*, and Supplementary Movie 2). For depth measurement, the slightly larger index of refraction in brain tissue (1.35–1.40 for the cortex (Bacallao et al. 2006; Binding et al. 2011) and as high as 1.47 for the white matter (Bacallao et al*.* 2006)), relative to water (~1.33), resulted in a slight underestimate (5–10%) of the actual imaging depth within the tissue, because the imaging depths reported here are the raw axial movement of the objectivelens.

We kept the maximum power at the brain surface to below 55 mW for all imaging sessions, but we still queried if our in vivo 3PM imaging may cause inflammation. We investigated cortical gliosis, a hallmark of inflammation, in mice imaged at different ages. Reactive GFAP+ astrocytes were found in the ipsilateral side of the juvenile cortex at both 3 and 17 dpi (Supplementary Fig. 2*A*). However, there were no obvious increases in Iba1 immunofluorescence (Supplementary Fig. 2*B*). Astrocyte activation was also noticed as increases in GFAP expression on the ipsilateral side to the craniotomy, without 3PM imaging, (Supplementary Fig. 2*C*). This is consistent with evidence that open-skull glass windows, as used here, activate cortical astrocytes (Xu et al. 2007). P3 and P8 pups after acute imaging with 3PM did not result in either reactive GFAP+ astrocytes (Supplementary Fig. 3*A*,*B*) nor in Iba1+ activated microglia (Supplementary Fig. 3*C*,*D*) in the cerebral cortex.

To provide an upper-bound estimate of the spatial resolution, we measured the lateral brightness distribution of small features within the juvenile mouse brain (Supplementary Fig. 4*A*) at 1039 μm depth. The full-widths at half maximum (FWHM) of the lateral brightness distributions was about 0.86 μm (Supplementary Fig. 4*B*). The axial FWHM value was approximately 4.45 μm in postnatal brain at 1371 μm depth, shown in Supplementary Fig. 4*C*,*D*.

**Figure 3:**
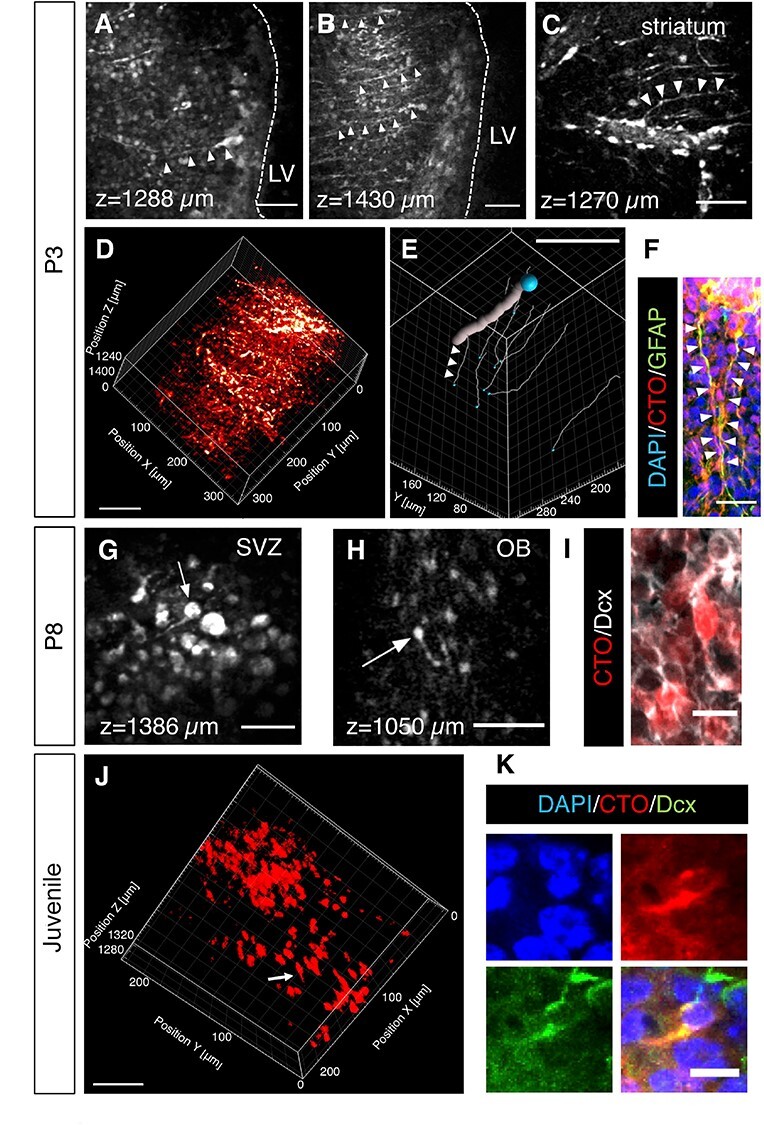
3PM imaging of SVZ and OB cell types. (*A*–*C*) Representative 3PM XY images of RGC like neural stem cells at different depths of P3 pups (2 dpi). Arrowheads indicate neural stem cells. (*D*) 3D reconstruction of white matter and SVZ from the depth of 1150–1472 μm of P3 pups (see Supplementary Movie 3). (*E*) Filament tracking in 3D reconstruction (*D*). The thick line indicates a blood vessel and the thin lines (ex. arrowheads) indicate individual cellular processes from different 3PM-imaged NSCs (see Supplementary Video 4). The blue balls indicate tracing starting points. (*F*) Immunostaining of GFAP in SVZ. The arrowheads indicate the basal processes of two neural stem cells labeled by both CTO and GFAP. (*G*, *H*) Representative 3PM XY images of neuroblasts at the depth of 1386 μm in SVZ (*G*) or 1050 μm in OB (*H*) of P8 pups (7 dpi). The arrows indicate neuroblasts. (*I*) Immunostaining of Dcx in OB confirming that CTO+ cells neuroblasts. (*J*) 3D reconstruction of the SVZ from the depth of 1271–1325 μm in a juvenile mouse (3 dpi; See Supplementary Video 5). The arrow points to a cell with migratory morphology. (*K*) Immunostaining showing a Dcx + cell in the SVZ labeled with CTO. Scale bars represent 50 μm in *A*, *B*, *C*, *G*, *H*, *J*; 80 μm in *D*; 100 μm in *E*; 20 μm in *F*; 10 μm in *I* and *K*.

**Figure 4:**
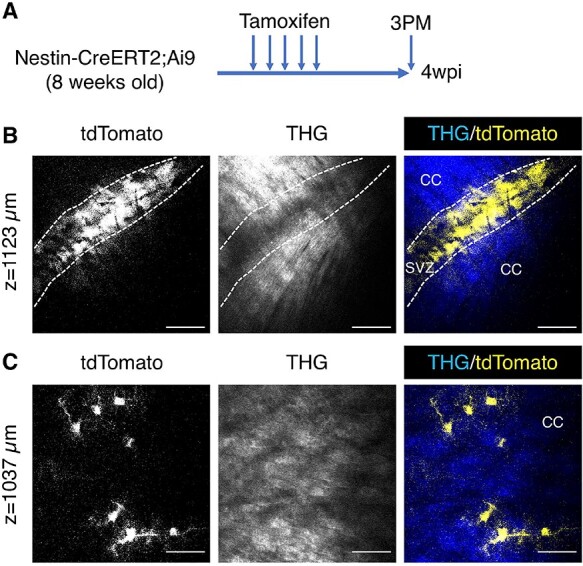
3PM imaging of Nestin-CreER2;Ai9. (*A*) A schematic of the experimental design. Imaging was carried out 4 weeks post injection (4 wpi). (*B*) A representative XY image of the SVZ at the depth of 1123 μm. (*C*) A representative XY image of the corpus callosum with fibrous astrocytes (yellow) at the depth of 1037 μm. Scale bars represent 50 μm in *B* and *C*.

To evaluate the cytoarchitecture of the postnatal SVZ, we examined CTO+ cells below the corpus callosum. Cells with elongated basal processes, with approximately 1 μm diameters, were observed at multiple depths (ex. 1288 and 1430 μm) (Fig. 3*A*,*B*). The cell bodies lined the LV wall, and their overall morphology resembled radial glial cells (RGC) which are postnatal SVZ stem cells. Importantly, when we followed the basal processes of the cells to the adjacent striatum, we found them in close proximity to blood vessels (Fig. 3*C*). This juxtaposition allows NSC to be controlled by extrinsic signals from the vascular niche (Mirzadeh et al. 2008; Shen et al. 2008). To confirm this interaction, we made 3D reconstructions showing the fine processes of NSC surrounding blood vessels (Fig. 3*D* and Supplementary Movie 3). Direct physical contact between these cells was further shown by filament tracking of representative processes (Fig. 3*E* and Supplementary Movie 4). Immunohistochemistry confirmed that the CTO+ cells with NSC morphologies described above expressed GFAP (Fig. 4*F*), a protein found in NSC (Ihrie and Alvarez-Buylla 2011).

To follow SVZ lineage progression, we next imaged pups at a later time point after CTO injection, that is, P8. Since TAPs are difficult to identify based on morphology, we chose to examine SVZ neuroblasts, which are small bipolar cells with distinctive leading processes (Doetsch et al. 1997). Cells with neuroblast morphology were found in the SVZ, for example at a depth of 1386 μm (Fig. 3*G*). CTO may label multiple cell types in the SVZ rather than revealing lineage progression. Therefore, to more definitively identify lineage progression, we imaged CTO+ neuroblasts generated in the SVZ that migrated into the olfactory bulb (OB) from the RMS. We focused on the OB-core RMS and detected CTO+ cells (Fig. 3*H*, Supplementary Fig. 5*A*). These 3PM-detected cells exhibited leading processes (Fig. 3*H*) and were co-stained with Dcx (Fig. 3*I*), which together support their neuroblast identity.

In the juvenile brain, CTO+ cells also exhibited morphological diversity (Fig. 3*J* and Supplementary Movie 5). An example of a 3PM-detected cell with the morphology of a unipolar neuroblast including a leading process is shown in Figure 3*J*. Another such cell is shown in Figure 3*K* with a CTO+ short process and positive Dcx staining showing neuroblast identity. Post-imaging immunohistochemical analysis confirmed various other SVZ niche cell types, S100β + ependymal cells (Supplementary Fig. 5*B*), Mash1+ TAPs (Supplementary Fig. 5*C*) and GFAP+ NSC (Supplementary Fig. 5*D*) and which were labeled by CTO at 3 days post injection (3 dpi) or 17dpi.

Conditionally inducible Nestin-CreERT2;Ai9 mice were then used to specifically label SVZ stem cells and their progeny (Lagace et al*.* 2007; Madisen et al. 2010). Expression of tdTomato was induced in NSC with tamoxifen injections, and imaging was performed 4 weeks later to analyze lineage progression (Fig. 4*A*). A dense population of tdTomato+ cells was observed in the SVZ, surrounded by THG labeled white matter (Fig. 4*B*). These experiments confirm the CTO data above and show that 3PM can be used to study live SVZ neurogenesis in this frequently used reporter mouse. Although the majority of SVZ NSC generate interneurons, which migrate to the OB, a small proportion of them contribute to astrocyte turnover in the corpus callosum (Yang et al. 2004; Sohn et al. 2015). Consistently, tdTomato+ cells were found in the white matter, for example at the depth of 1037 μm (Fig. 4*C*). Unlike bushy protoplasmic astrocytes, these cells exhibited fewer but thicker branches (Fig. 4*C*), a feature of fibrous astrocytes in the white matter (Robel et al. 2011).

**Figure 5:**
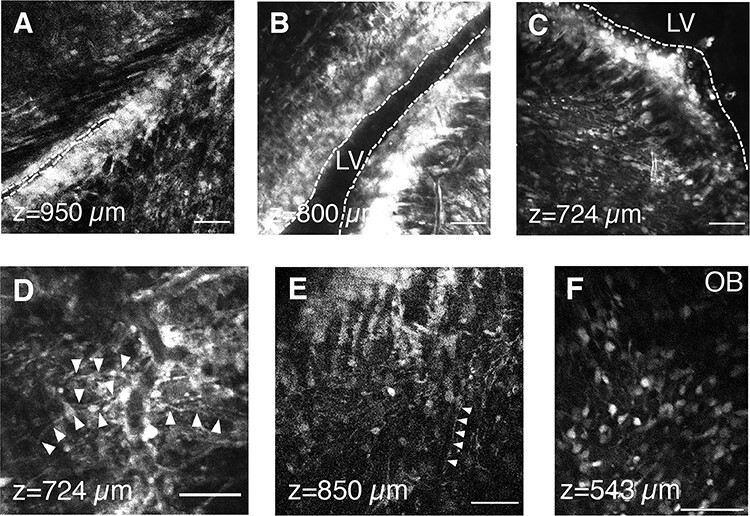
3PM imaging of SVZ and OB with CellTracker Green. (*A*–*C*) Representative 3PM XY images of the SVZ and LV at different depths at 2 dpi. LV indicated by dotted line. (*D*–*E*) Representative 3PM XY images of neural stem cells. The interaction between neural stem cells and blood vessels is shown in (*D*). Arrowheads indicate processes of cells with NSC-like morphology. (*F*) Representative 3PM XY image of olfactory bulb at the depth of 543 μm. This figure was generated from data generated at Oxford University. Scale bars represent 50 μm in *A*–*F*.

To further examine the application of 3PM in SVZ imaging, we utilized another custom-built 3PM in Oxford. Postnatal pups were injected with CellTracker Green (CTG) as before and imaged 2–3 dpi (P3–4). We acquired stacks to a maximum depth of around 950 μm (Supplementary Movie 6). CTG fluorescent signals were found throughout the stack with the resolution to distinguish various cellular morphologies. In particular, bright and dense bands of SVZ cells next to the LV were detected at different depths (Fig. 5*A*–*C*). Moreover, radial glia-like cells were detected within these areas (Fig. 5*C*,*E*). Similar to our experiments with CTG described above, we found physical interactions between blood vessels and cells with morphology corresponding to NSC (Fig. 5*D*). In the OB at 5 dpi (P6), CTG-labeled neuroblasts, with diverse morphologies, were also visible at multiple depths (ex. 543 μm) (Fig. 5*F*).

## Discussion

In summary, we demonstrated the utility of 3PM to visualize the murine SVZ in postnatal and juvenile mice. We labeled SVZ cells with dyes as well as genetically, and both were compatible with 3PM. SVZ cell type identification has been a major challenge for live imaging (Nam et al*.* 2007; Lacar et al. 2010), but here with 3PM, we successfully identified multiple cell types based on morphological analysis and anatomical position in live mice. Our results from post-hoc immunohistochemistry and multiple labeling techniques show good morphological concordance between 3PM and histological or ex vivo detection of morphological SVZ cell types. This study demonstrates the clear power and potential of three-photon microscopy that can be harnessed by biomedical researchers using readily available commercial hardware. Specifically, 3PM allows greater depth of live imaging than does 2PM.

We were able to image the SVZ of live mice to depths >1400 microns from the brain’s surface, a depth much greater than the reported limits of circa 500 microns (Theer and Denk 2006) to 850 microns with 2PM (Liu et al. 2019). This limitation precludes study of the SVZ in live mice with 2PM and investigators have thus relied on imaging slices (Nam et al*.* 2007; James et al*.* 2011). The dorsal-most SVZ is approximately 1000–1500 microns from the surface of the brain, depending on the age of the mouse. To compensate for the depth of the other neurogenic niche, the subgranular zone, scientist have made cortical lesions and lowered lenses into them and used the method to study lineage progression (Pilz et al*.* 2016, 2018). Whereas they have collected remarkable images, the cortical lesion may have altered the behavior of the stem cells. Our method does not necessitate removal of overlying cortical tissue, is thus less damaging and may allow a more naturalistic representation of the stem cell niche.

3PM requires robust advanced laser sources which are now available from commercial suppliers (e.g., from Coherent and Spectra-Physics). The optical parametric amplifier systems used provided the correct combination of short-pulse duration, repetition rate and pulse energy required for effective imaging. Microscopes compatible for long wavelength imaging, and objective lenses with long working distance and high numerical aperture, are also available from several manufacturers. The imaging performance at depth can be further improved by using adaptive optics (AO) (Booth 2007; Ji et al. 2010). While low-order wavefront compensation with AO cannot significantly extend the imaging depth, and AO adds complexity in the imaging setup, it will be helpful for visualizing small features such as neuronal processes at depth.

We used GFAP and Iba1 immunohistochemistry as these markers label astrocytes and microglia, two cell types that are sensitive to most brain damage. Microglia respond to brain injury very rapidly and astrocytes typically lag behind by a day or two. Similar to what others have found (Xu et al*.* 2007), we showed increased GFAP expression in the top layers of the cerebral cortex around the craniotomy, and this provided a useful positive control showing such increases could be detected. In contrast, we did not find increases in Iba1 expression in the same regions of the cortex suggesting that inflammation was minimal. Importantly, we also did not find any changes in either GFAP or Iba1+ immunofluorescence in and around the SVZ, the area imaged. This suggests that 3PM imaging did not induce photodamage in the stem cell niche. We cannot rule out that inflammation or phototoxicity would appear at time points after 3PM imaging later than those used in this study. We also cannot completely rule out that subtle damage without actual cell death occurred in our imaging studies. For example, DNA damage or cell membrane disruption may have occurred during or after 3PM imaging. Future studies of these potential events as well as different forms of cell death such as apoptosis, necroptosis or ferroptosis would help confirm our findings.

In conclusion, we have developed a minimally damaging approach to imaging the live SVZ in postnatal and juvenile mice. This 3PM approach is well suited for imaging SVZ cell dynamics of stem cell activation, proliferation and migration and could be used over several hours to visualize fast events such as calcium dynamics, neuroblast migration, microglial activation, and over days to weeks in the same animals to visualize slow events such as stem cell activation, lineage progression, and attempts at regeneration. Similar to (Pilz et al*.* 2018), 3PM could be used over several days for imaging clonal evolution and also the impacts of aging on the SVZ. We plan to use 3PM for imaging combined with the study of molecular mechanisms and the response of the SVZ to models of disease, such as gliomagenesis (Bardella et al*.* 2016).

## Supplementary Material

Video_1_bhab400

Movie_2_bhab400

Movie_3_bhab400

Movie_4_bhab400

Movie_5_bhab400

Video_6_bhab400

Movie_legends_bhab400

Suppl_Fig_1_bhab400

Suppl_Fig_2_bhab400

Suppl_Fig_3_bhab400

Suppl_Fig_4_bhab400

Suppl_Fig_5_bhab400

## Notes

We thank Christian Wilms (Scientifica) and Marcus Beutler (APE) for scientific discussion, and APE for the parts for the pulse compressor in Oxford. *Conflict of Interest*: None declared.
