# Supplementary material for: Intravital imaging of the murine subventricular zone with three photon microscopy
Source: Cereb Cortex. 2022 Jan 14;32(14):3057–67. doi: 10.1093/cercor/bhab400 (PMC9290563; doi:10.1093/cercor/bhab400)
Supplement: Movie_legends_bhab400 [file movie_legends_bhab400.pdf]

Movie 1
The movie contains 512 x 512 pixel images of fluorescent signals from CTO in a P3 brain. Depth in µm is shown on bottom left corner. Scale bar, 50 µm.

Movie 2
The movie contains fluorescent signals from CTO (red) and THG signals (green). The 3D reconstruction shows the depth from 1050 µm to 1400 µm in a juvenile brain. Scale bar, 100 µm.

Movie 3
The movie contains fluorescence signals from CTO (red). The 3D reconstruction shows fine processes of cells at depths from 1150 µm to 1472 µm in a P3 brain. Scale bar, 80 µm.

Movie 4
Filament tracking in a P3 brain. The 3D reconstruction shows thin lines that represent the fine basal processes of neural stem cells and the thick line represents a blood vessel. One of the processes has a direct contact with the blood vessel. Scale bar, 100 µm.

Movie 5
The movie contains fluorescence signals from CTO (red). The 3D reconstruction shows the depth from 1271 µm to 1325 µm in a juvenile brain. Scale bar, 50 µm.

Movie 6
The movie shows CTG imaging with our 3PM at U Oxford. Cells are shown from depths of from 761 to 950 µm in a P3 brain. Scale bar, 50 µm.
