# Supplementary material for: Intravital imaging of the murine subventricular zone with three photon microscopy
Source: Cereb Cortex. 2022 Jan 14;32(14):3057–67. doi: 10.1093/cercor/bhab400 (PMC9290563; doi:10.1093/cercor/bhab400)
Supplement: Suppl_Fig_1_bhab400 [file suppl_fig_1_bhab400.pdf]

Supplementary Material

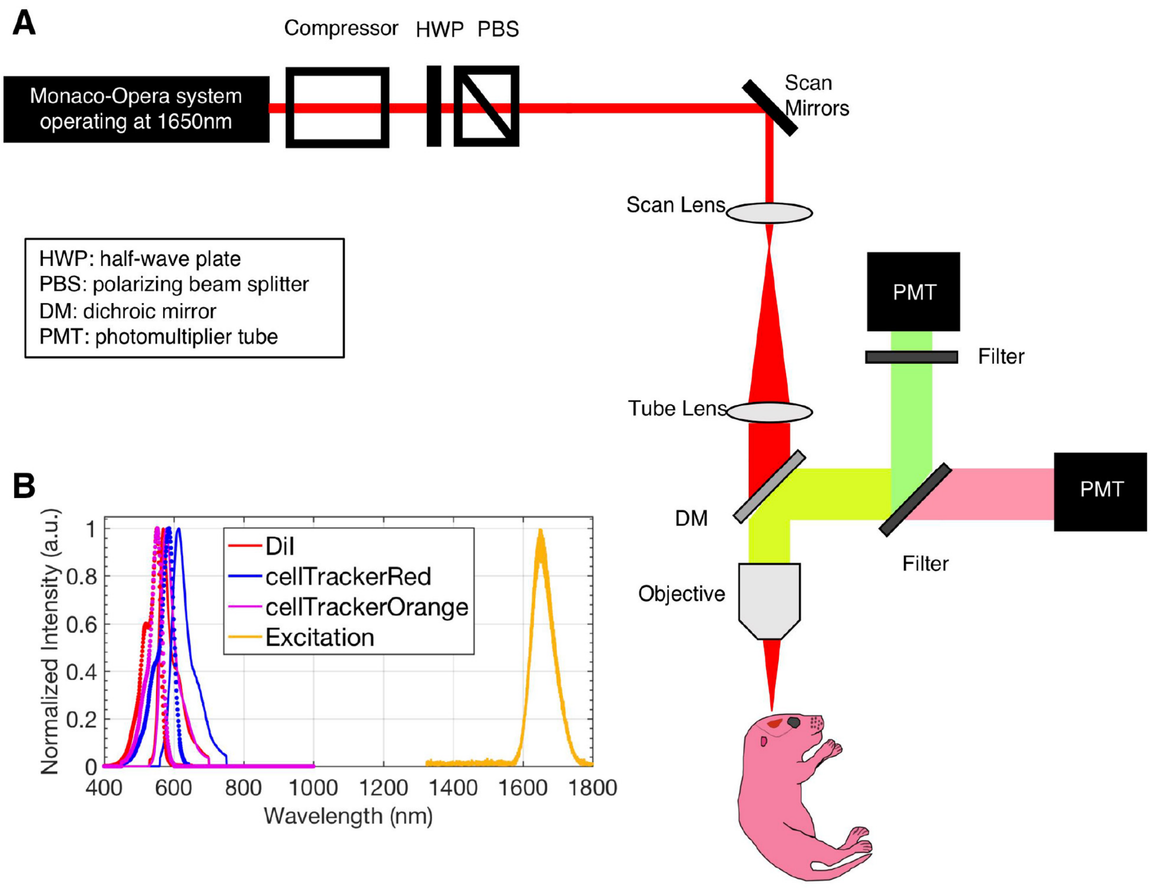

Fig. S1 3PM experimental setup
(A) Main elements of the 3-photon microscope used at Cornell University.
(B) The spectra of the laser (yellow) and excitation (dashed lines) and emission (solid lines) spectra of DiI, CellTracker Red (CTR), and CellTracker Orange (CTO) used for 3-photon imaging. Data for the fluorescent dyes were obtained from Life Technologies, USA.
