# Supplementary material for: Intravital imaging of the murine subventricular zone with three photon microscopy
Source: Cereb Cortex. 2022 Jan 14;32(14):3057–67. doi: 10.1093/cercor/bhab400 (PMC9290563; doi:10.1093/cercor/bhab400)
Supplement: Suppl_Fig_2_bhab400 [file suppl_fig_2_bhab400.zip › Suppl_Fig_2_bhab400.pdf]

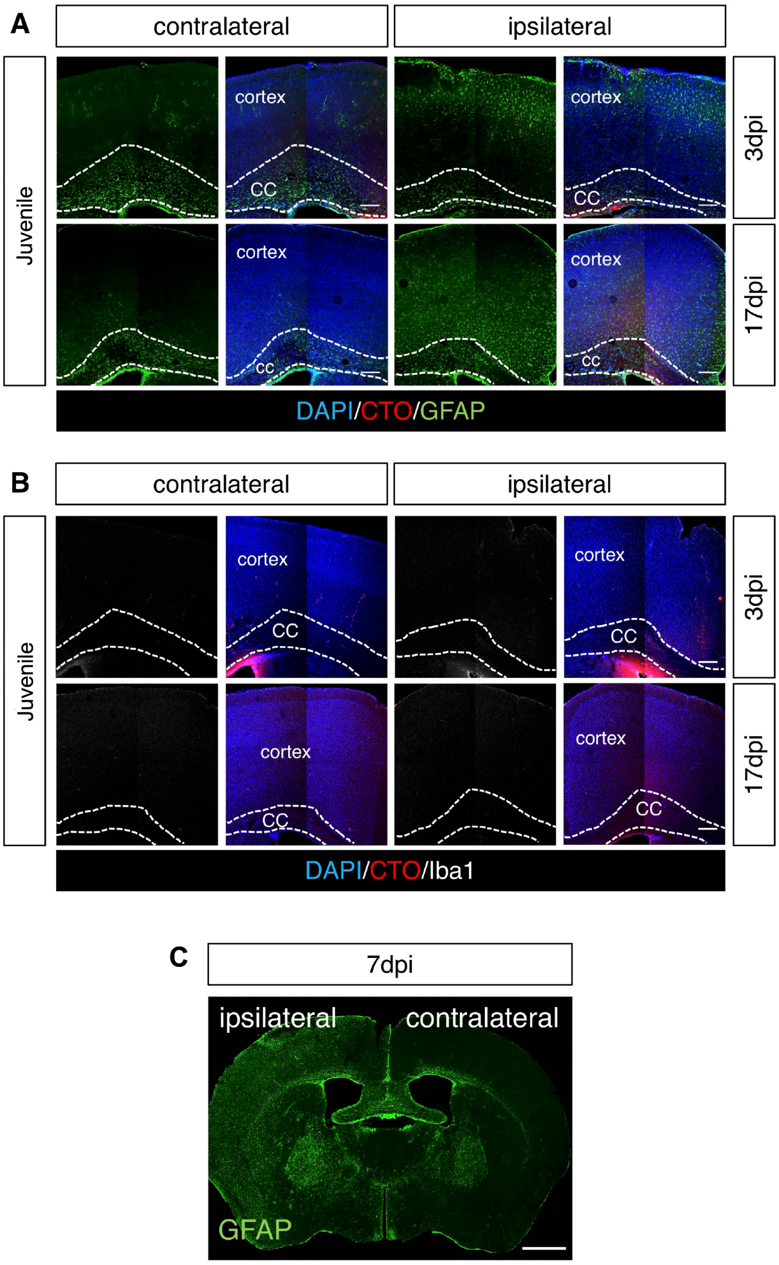

Fig. S2 Gliosis analysis in the cerebral cortex of juvenile mice
(A) Immunohistochemistry of GFAP in juvenile brain sections, imaged and fixed 3 days or 17 days post injection.
(B) Immunohistochemistry of Iba1 in juvenile brain sections, 3 days or 17 days post injection.
(C) Immunohistochemistry of GFAP in juvenile brain sections from mice with craniotomy surgery, but which did not have 3PM imaging.
Scale bars represent 200 μm in A and B. Scale bar represents 1000 μm in C.
