# Supplementary material for: Intravital imaging of the murine subventricular zone with three photon microscopy
Source: Cereb Cortex. 2022 Jan 14;32(14):3057–67. doi: 10.1093/cercor/bhab400 (PMC9290563; doi:10.1093/cercor/bhab400)
Supplement: Suppl_Fig_3_bhab400 [file suppl_fig_3_bhab400.zip › Suppl_Fig_3_bhab400.pdf]

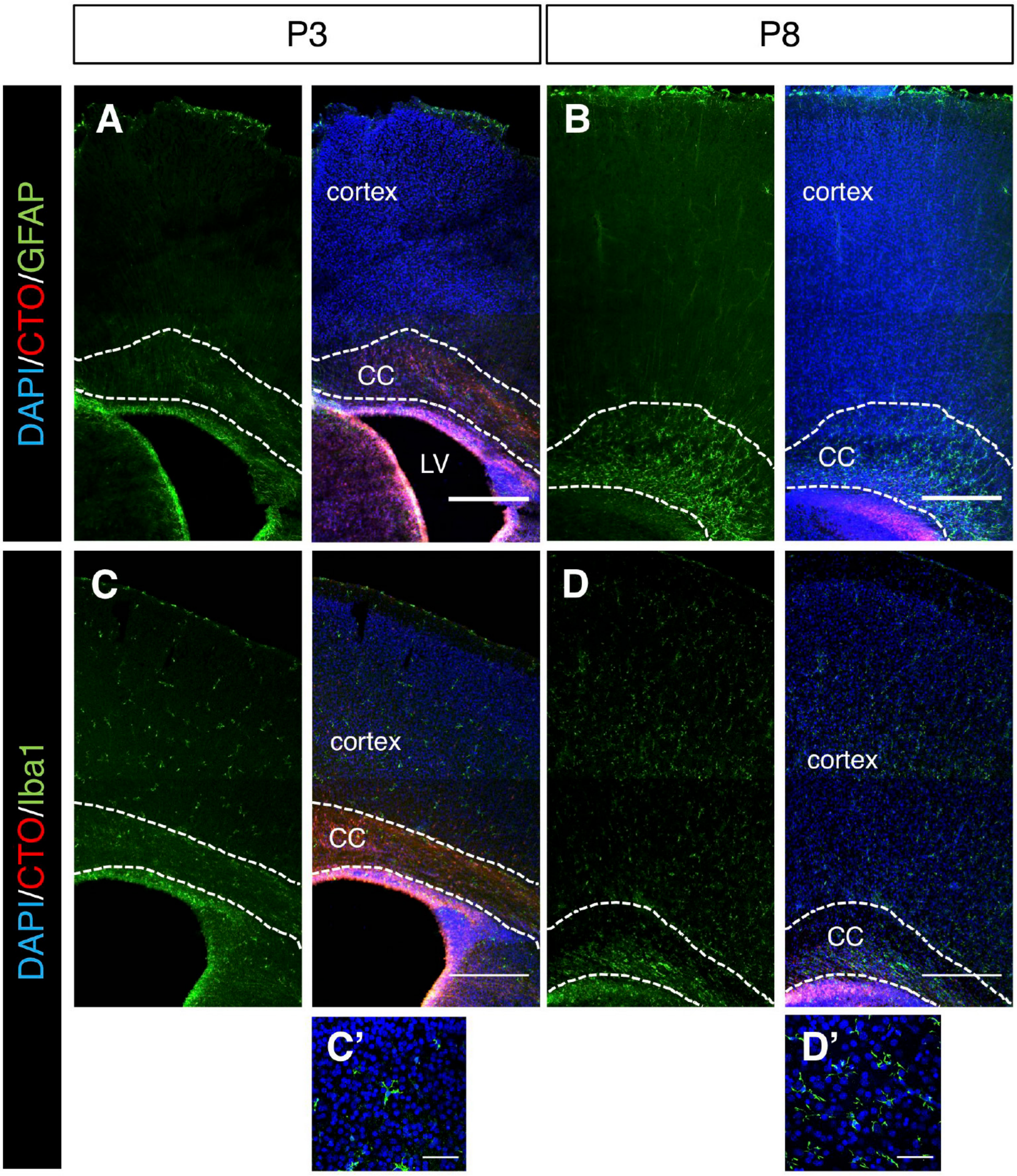

Fig. S3 Gliosis analysis in the ipsilateral cortex of 3PM imaged postnatal pups
(A-B) Immunohistochemistry of GFAP in P3 and P8 brain sections.
(C-D) Immunohistochemistry of Iba1 in P3 and P8 brain sections. c' and d' are high magnification images of Iba1+ cells in cortex in c and d, respectively.
Scale bars represent 300 μm in A, B, C, and D; 100 μm in C' and D'.
