# Supplementary material for: Intravital imaging of the murine subventricular zone with three photon microscopy
Source: Cereb Cortex. 2022 Jan 14;32(14):3057–67. doi: 10.1093/cercor/bhab400 (PMC9290563; doi:10.1093/cercor/bhab400)
Supplement: Suppl_Fig_4_bhab400 [file suppl_fig_4_bhab400.pdf]

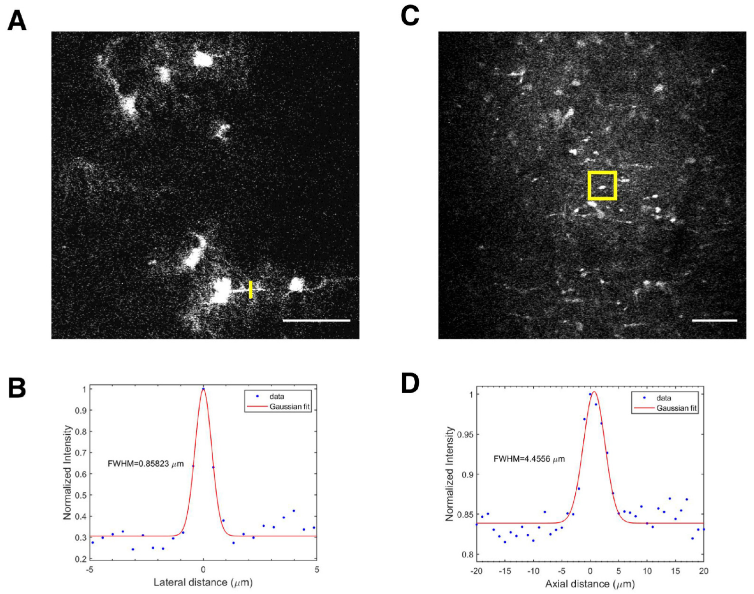

Fig. S4 Intensity profiles of subcellular structures

(A-B) The lateral brightness distribution of small features within the juvenile mouse brain (A) fine astrocyte processes are visible at 1039 μm depth and the FWHM of the lateral brightness distributions (B).

(C-D) The axial brightness distribution of small features of cells corresponding with radial glia processes within the postnatal mouse brain (C) at 1371 μm depth and the FWHM of the axial brightness distributions (D).

Scale bars represent 50 μm in A and C.
