# Supplementary material for: Intravital imaging of the murine subventricular zone with three photon microscopy
Source: Cereb Cortex. 2022 Jan 14;32(14):3057–67. doi: 10.1093/cercor/bhab400 (PMC9290563; doi:10.1093/cercor/bhab400)
Supplement: Suppl_Fig_5_bhab400 [file suppl_fig_5_bhab400.zip › Suppl_Fig_5_bhab400.pdf]

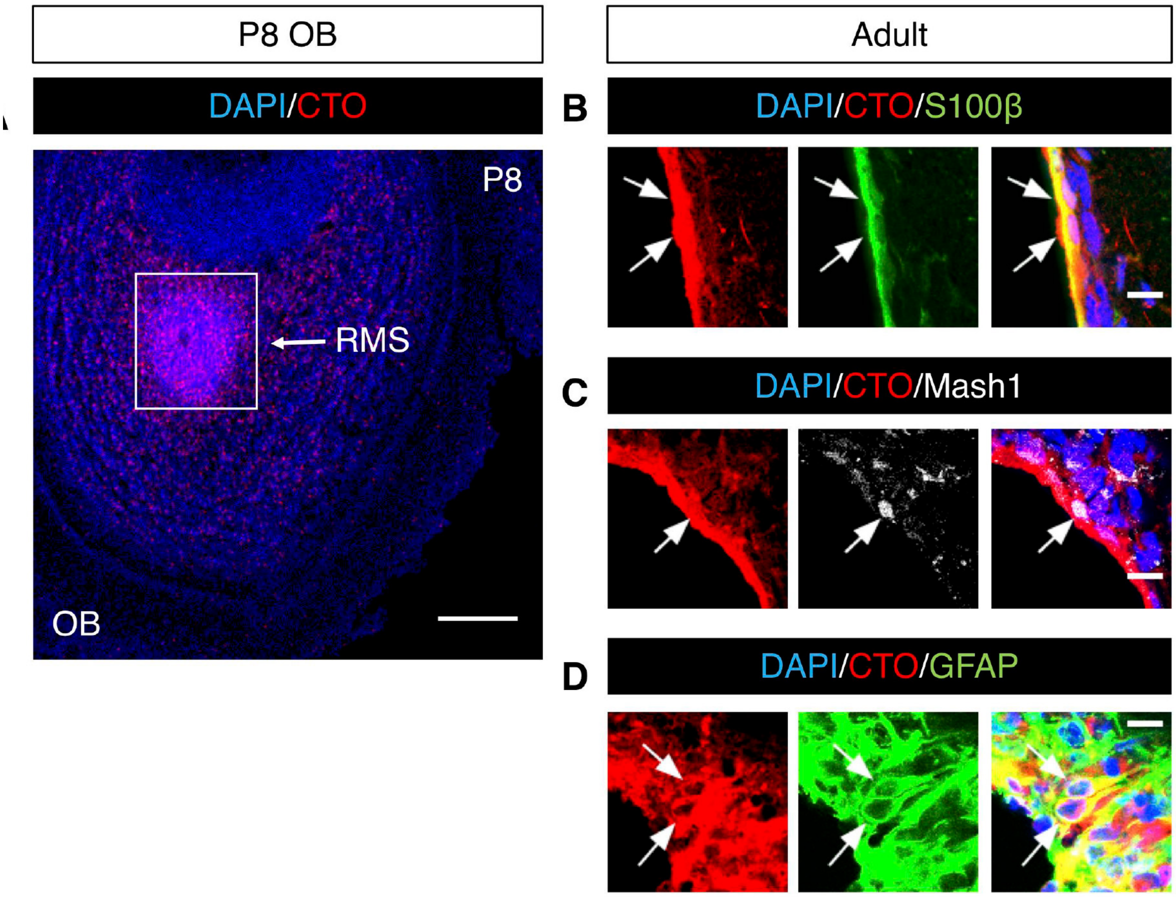

Fig. S5 Cell type identification of CTO-labelled cells in OB and SVZ

(A) Confocal imaging of a representative brain section labelled with CTO (red) in the RMS extending into the P8 OB.

(B) Immunostaining of S100β in the SVZ. Arrows indicate ependymal cells.

(C) Immunostaining of Mash1 in the SVZ. The arrow indicates a transit amplifying progenitor.

(D) Immunostaining of GFAP in the SVZ. Arrows indicate the neural stem cells. (B-D) Juvenile mice.

Scale bars represent 200 μm in A; 10 μm in B, C, D.
